# Dual stressors of infection and warming can destabilize host microbiomes

**DOI:** 10.1098/rstb.2023.0069

**Published:** 2024-05-06

**Authors:** J. D. Li, Y. Y. Gao, E. J. Stevens, K. C. King

**Affiliations:** ^1^ Department of Biology, University of Oxford, Oxford OX1 2JD, UK; ^2^ Shenzhen Branch, Guangdong Laboratory of Lingnan Modern Agriculture, Genome Analysis Laboratory of the Ministry of Agriculture and Rural Affairs, Agricultural Genomics Institute at Shenzhen, Chinese Academy of Agricultural Sciences, Shenzhen, Guangdong 518120, People's Republic of China; ^3^ School of Ecology and Nature Conservation, Beijing Forestry University, 35 Tsinghua East Road, Beijing 100083, People's Republic of China; ^4^ Department of Zoology, University of British Columbia, Vancouver, V6T 1Z4, Canada; ^5^ Department of Microbiology & Immunology, University of British Columbia, Vancouver, V6T 1Z3, Canada

**Keywords:** microbiomes, parasite, *C* *. elegans*, global climate change

## Abstract

Climate change is causing extreme heating events and intensifying infectious disease outbreaks. Animals harbour microbial communities, which are vital for their survival and fitness under stressful conditions. Understanding how microbiome structures change in response to infection and warming may be important for forecasting host performance under global change. Here, we evaluated alterations in the microbiomes of several wild *Caenorhabditis elegans* isolates spanning a range of latitudes, upon warming temperatures and infection by the parasite *Leucobacter musarum*. Using 16S rRNA sequencing, we found that microbiome diversity decreased, and dispersion increased over time, with the former being more prominent in uninfected adults and the latter aggravated by infection. Infection reduced dominance of specific microbial taxa, and increased microbiome dispersion, indicating destabilizing effects on host microbial communities. Exposing infected hosts to warming did not have an additive destabilizing effect on their microbiomes. Moreover, warming during pre-adult development alleviated the destabilizing effects of infection on host microbiomes. These results revealed an opposing interaction between biotic and abiotic factors on microbiome structure. Lastly, we showed that increased microbiome dispersion might be associated with decreased variability in microbial species interaction strength. Overall, these findings improve our understanding of animal microbiome dynamics amidst concurrent climate change and epidemics.

This article is part of the theme issue ‘Sculpting the microbiome: how host factors determine and respond to microbial colonization’.

## 1. Introduction

Global climate change has led to multiple climate hazards including more extreme temperatures, resulting in population decline and biodiversity loss [1]. Shifting global temperatures is also changing the geographical distribution of infectious diseases [2,3]. As temperatures increase, hosts and parasites are experiencing shifts in their thermal environment, driving variation in disease outcomes [4,5]. Thus, projections of species persistence in the changing world will need to account for threats posed by both warming and infection, as well as the interaction of these dual stressors [6,7].

Accumulating evidence shows that host health in the face of changing temperatures can be mediated by host microbiomes [8,9] and parasite infection [10,11]. Microbiomes are highly sensitive to biotic or abiotic disturbances [12–14]. Changes in microbiome structure and stability, as a result, are increasingly recognized as meaningful indicators of altered host health [12,13,15]. Studying microbiome dynamics under warming and infection scenarios provides important predictions of species persistence under climate change and infectious diseases [12,14,16].

Temperature and parasite infection can both disrupt host microbiome structure [13,17–23]. Across animal species, experimental warming has been shown to decrease host microbiome phylogenetic diversity and alter microbiome composition [18]. The effects of temperature on host microbiota can vary depending on local environmental conditions. Hosts adapted to more variable thermal conditions can experience less microbiome diversity loss under thermal stress [18]. Whilst infection can alter microbiome diversity, the direction of change varies across host and parasite species. For example, *Clostridioides difficile* infection in the human gut can decrease microbiome diversity [19], while *Mycobacterium tuberculosis* infection has the opposite effect [20,21]. Higher temperatures (prior to *Batrachochytrium dendrobatidis* exposure) and infection individually decreased skin microbiome richness on red-backed salamanders (*Plethodon*) [13]. The extent to which warm temperatures and infection might interact to structure host microbiomes more extensively is unclear.

Here, we explored the separate and combined effects of infection and warming (i.e. at different time points across host lifespan) on host microbiome structure and stability. We used *Caenorhabditis elegans* nematodes, representative species of their natural gut microbiome (CeMbio community isolated from temperate nematodes [24]), and a natural parasite of *Caenorhabditis* spp., *Leucobacter musarum* [25]. Use of *C. elegans* with a consortium of culturable bacterial associates enabled us to explore metabolic interactions with the parasite. We included a diversity of wild nematode isolates across a range of latitudes to understand the impact of habitat adaptation on these relationships. The fitness levels of *C. elegans* isolates can be dependent on thermal preferences [26], with phenotypes exhibited varying across temperatures and life stages [27]. We predicted that warming and infection might individually destabilize host microbiomes, with the extent of disruption dependent on the timing of warming. Destabilization induced by warming and infection could be characterized by increased inter-individual variability in microbiome structure (or dispersion). Such a pattern could indicate a loss of host ability to regulate community composition in an otherwise stable state [28–30]. We used 16S rRNA sequencing and metabarcoding analysis, to measure changes in microbiome diversity and dispersion. We established microbial species co-occurrence networks to assess the strength and direction of species associations at different times when warming occurred. We reconstructed species-level genome-scale metabolic models using whole-genome sequences to explore the potential metabolic interactions between microbiome species and the parasite. Overall, we found that host microbiomes were destabilized in a non-additive fashion by the stressors. The timing of warming and degree of laboratory adaptation (laboratory versus wild isolates) played a role on microbiome responses to both stressors. These results highlight the dynamic nature of host microbiomes in a more thermally variable and infectious world.

## 2. Material and methods

### (a) Nematode, bacterial strains and maintenance

We used the laboratory-adapted N2 and eight wild *C. elegans* isolates (obtained from the Caenorhabditis Genetics Centre; CGC, Minnesota, USA) originally collected across a range of latitudes (see the electronic supplementary material, table S1 for list of isolates and their locations of origins). N2 has been commonly used in biological research since its introduction to the research community by Sydney Brenner in 1974 [31]. The wild isolates were chosen from a range of latitudes at similar elevations. At the start of all experiments, *C. elegans* isolates were thawed from frozen stocks and maintained at 20°C, according to a standard maintenance protocol using nematode growth medium (NGM) plates seeded with *Escherichia coli* OP50 as food [32]. OP50 was grown at 30°C overnight in Luria-Bertani (LB) broth, with 100 µl of culture spread onto each NGM plate and incubated at 30°C overnight. Worm populations were synchronized and made sterile by bleaching [32].

For host microbiome colonization, we used a community of 12 bacterial isolates found naturally associated with *C. elegans* (CeMbio kit) [24]. The CeMbio community is a simplified natural microbiome derived from a meta-analysis on wild *C. elegans* [33]. Each species is readily culturable, with its full genome sequenced [24]. CeMbio strains can colonize the worm gut individually or comprise a robust community during host development and potentially affect nematode life history [24]. Each CeMbio strain was grown individually in LB broth for 24–48 h at 25°C. Cultures were then standardized to an optical density (OD; 600 nm) of 1 for consistent doses within and across experiments. The community inoculum was prepared by mixing equal volumes of each bacterial strain. Microbiome exposure plates were prepared by spreading 400 µl of the mixture inoculum onto 9 cm NGM plates. For comparison, the OP50 feeding plates were prepared by spreading the same amount of culture (standardized to the same OD as microbiome cultures). We ensured individual CeMbio strains could colonize the worm gut during host development. We exposed *C. elegans* to each CeMbio strain (the same dose as in the community mixture), collected and crushed pre-adult worms for DNA extraction, and performed standard polymerase chain reaction (PCR) using both general and species-specific primers to detect the presence of individual strains.

We exposed nematodes to *L. musarum* sp. nov. subsp. *musarum* subsp. nov strain CBX152T (*L. musarum*), a highly virulent parasite isolated from *Caenorhabditis tropicalis* in Cape Verde [34]. *Leucobacter musarum* causes severe rectal disease and ultimately death in *C. elegans* [34]*.* Other species of this genus have been found naturally infecting *C. elegans* [35,36]*.* For parasite exposures, *L. musarum* was grown in LB broth at 30°C overnight, and the culture was standardized to OD(600) 0.3. The infection exposure plates were prepared by streaking 74 µl of inoculum containing 20% *L. musarum* and 80% OP50 (standardized to OD(600) 1) onto 5.5 cm NGM plates and incubating at 25°C for 24 h [36]. Control plates were prepared by spreading a similar amount of OP50 culture.

### (b) Sampling of *Caenorhabditis elegans* gut microbiomes

**Figure 1.**
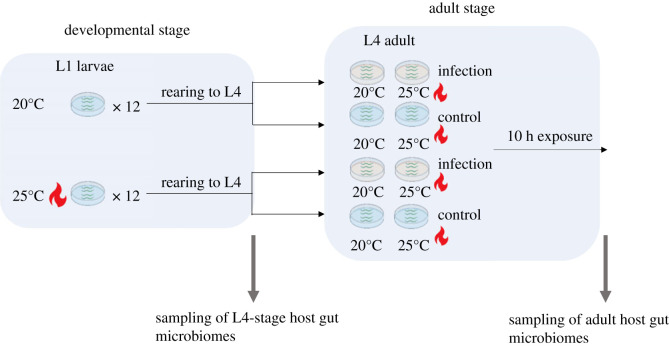
Schematic of experimental microbiome sampling. In brief, L1 stage laboratory-adapted N2 worms or wild worm isolates were grown on microbiomes at 20°C or 25°C until the L4 stage. L4 worms were exposed to parasites (or not) at 20°C or 25°C. In total, four temperature regimes were used: 20°C–20°C (ambient temperatures during development and adulthood), 20°C–25°C, 25°C–20°C and 25°C–25°C. Host gut microbiomes were sampled pre-adulthood (from L4 young adults) and at adulthood.

We manipulated temperature during the worm developmental period (from L1 to L4 young adults) and subsequent parasite exposure (figure 1). We used the ambient temperature of 20°C and a warmer temperature of 25°C. This higher temperature causes mild heat stress for temperate *C. elegans* isolates with the potential to shorten lifespan and reduce reproductive output [37,38].

To study host microbiome dynamics under infection and warming, approximately 1000 L1 nematodes were grown on OP50 or microbiome feeding plates at either 20°C or 25°C for approximately 48 h or approximately 34 h, until they reached L4 (worms develop faster at higher temperatures). L4 young adults were transferred to infection (20% *L. musarum* and 80% OP50) or control (OP50 only) plates, and left for 24 h at either 20°C or 25°C. Each treatment was replicated six times. Worm populations were sampled before they were transferred to infection plates (labelled ‘pre-adult’) and after 10 h (labelled ‘adult’) under all temperature treatments. We sampled worm microbiomes at 10 h post-parasite exposure to ensure that host microbiomes were sampled from mostly live, infected hosts [36]. To collect host gut microbiomes, we washed worms off the NGM plate using M9 buffer. Approximately 700 worms were harvested from each replicate and crushed using the QIAGEN TissueLyser II for 5 min to release gut microbiomes. We collected microbiomes from 60 N2 worm samples and 80 wild isolate samples. Collected bacterial samples were immediately frozen at −20°C.

### (c) Amplicon sequencing and data processing

DNA was extracted from frozen bacterial samples using the ZymoBIOMICS DNA Miniprep kit (Zymo) according to manufacturer's instructions. DNA extractions were conducted in a random order to avoid batch effects on downstream microbiome data. We also extracted DNA from the frozen stock of *in vitro* CeMbio community culture that was used for host exposure, as a reference community, to compare with host gut microbiomes. The V3-V4 regions of bacterial 16S rRNA were amplified using the universal primer pair 341F (5′-CCTACGGGNGGCWGCAG-3′) and 805R primer (5′-GACTACHVGGGTATCTAATCC-3′). PCR amplicons were sequenced on the Illumina Miseq platform using 2 × 300 bp v3 chemistry (Integrated Microbiome Resources, Canada [39]).

FastQC [34] and MultiQC [40] were used for initial visualization of read quality, primers were removed using Cutadapt [41]. Paired-end reads were joined using vsearch [42]. All low-quality reads were then filtered using default quality thresholds before starting the Deblur [43] workflow to denoise and classify sequences into amplicon sequence variants (ASVs). Trimming length was determined as 400 bp after manually viewing the quality plot. As full-length 16S rRNA sequences for CeMbio strains were well-characterized [24], we processed the obtained sequencing reads through the closed-reference operational taxonomic unit picking pipeline in QIIME2 [44]. To build the reference, we downloaded full-length 16S sequences for all CeMbio strains and converted sequences to qza formatted reference files for processing by QIIME2. Taxonomy of the resolved ASVs was assigned by clustering ASVs to the customized reference with 99% similarity thresholds.

### (d) Microbiome diversity and compositional analysis

To account for the different number of reads in each sample, we used a normalization method called scaling with ranked subsampling (SRS) [45]. This method can better preserve the original microbial community structure and minimize subsampling errors compared with the rarefy approach. After normalization, five samples from the wild isolates assay were discarded because of low sequencing depth. Based on SRS-normalized data, four alpha diversity indices—Shannon's index, richness, evenness, and Faith's phylogenetic distance (Faith's PD)—were computed using the R package phyloseq [46]. These four alpha diversity metrics individually quantified a different aspect of community diversity [47]. Richness is the observed number of different species in the microbial community, which does not consider species abundance. Evenness measures the equity in species abundance in the community, where bigger values represent more evenly distributed species abundance. Shannon's index quantifies the uncertainty in predicting the species identity of an individual taken randomly from the community, which considers both species richness and evenness. Faith's PD is the only metric accounting for species phylogenetic distance in the community. It is measured as the sum of branch lengths between the observed species on a phylogenetic tree.

To quantify the dominance level of microbial communities, three different dominance indices—Berger-Parker, McNaughton's, and Simpson's indices—were calculated using the R package mia [48]. The Berger-Parker index is the relative abundance of the most abundant species in the community, and McNaughton's index is the sum of relative abundances of the two most abundant species in the community. Simpson's index is the probability that two randomly chosen species are the same. All three range from 0 to 1, where bigger values represent greater dominance.

For beta diversity, community distance matrices of unweighted and weighted UniFrac, Bray-Curtis and Aitchison dissimilarity were calculated based on either SRS-normalized relative abundance or SRS-normalized read counts, using the R package phyloseq. Different beta diversity metrics can lead to variable study power, thus consistency across multiple metrics indicates strong and more reliable patterns [49]. Permutational analysis of variance (PERMANOVA) was conducted with 9999 replications on each distance metric to evaluate differences in microbiome structure and composition between treatments using the R package vegan [50]. To test microbial stability and dispersion at a multivariate level, for each beta diversity metric, we calculated pairwise distance of samples, representing the distance of two samples within the same treatment group. Sets of values were compared between treatment groups using a Wilcoxon rank-sum test.

### (e) Bacterial co-occurrence analysis

Microbial co-occurrence networks are commonly built from species abundance data. They are widely applied to explore interactions and co-existence of bacterial species [51]. In the co-occurrence network, nodes are the bacterial species, and links between nodes usually represent significant species associations [51]. Links can have different weights which indicate varying association strength. Positive links (i.e. higher abundance of bacterial A associated with higher abundance of bacterial B) indicate facilitative interactions, and negative links (i.e. higher abundance of bacterial A associated with lower abundance of bacterial B) indicate competitive interactions [51].

We established microbial co-occurrence networks on the species level for different treatment groups using SparCC [52] program wrapped in SpiecEasi R package [53]. This program is robust to any distribution of community abundances. One-hundred bootstrap replicates were used to calculate significance levels, the threshold for SparCC correlation matrix was set at 0.3 and spurious links (absolute correlation coefficient <0.3) were removed. Links with *p*-values <0.05 were considered significant correlations. The number of positive and negative links was summarized for each treatment group.

### (f) Test for differentially abundant species

We performed differential species analysis using ALDEx2 (a clr based method) [54]. A non-parametric Wilcoxon rank-sum test was conducted on each of the species between treatment groups using aldex.ttest in R. Species with a Benjamini & Hochberg adjusted *p*-value < 0.05 were considered to be differently enriched between groups. The expected value of group distribution difference (median log2 difference) and pooled group variance (median log2 dispersion) were calculated using the aldex.effect function. So was the standardized effect size on species abundance difference between groups. Species with an effect-size of >0.3 were considered to have a large difference between groups. Further exploration between species enrichment and their potential metabolic interactions and phylogenetic distance were conducted (electronic supplementary material, Methods and Results).

### (g) Statistical analysis

Unless specified, all analyses were conducted in R 4.1.0 (RStudio 2023.03.1 + 446). Data were assessed for normality using the shapiro.test function in R prior to a *t*-test or non-parametric Wilcoxon rank-sum test. To test differences between more than two levels, an ANOVA or Kruskal Wallis test was used. We compared microbiome alpha, beta diversity and beta dispersion between infected versus uninfected hosts, as well as between hosts under different warming regimes. Comparison was also made between pre-adult and uninfected adult host microbiomes, which reflected the temporal change of pre-adult host microbiomes after 10 h off the environmental microbiomes. The full list of hypotheses tested and detailed statistical results are shown in the electronic supplementary material, table S11.

## 3. Results

We generated a total of 2 014 429 high-quality sequences (average length 400 bp) from 142 microbiome samples (including 140 *in vivo* samples, and two *in vitro* CeMbio community cultures). We found that CeMbio microbiomes can colonize pre-adult worm guts together, across the laboratory and wild isolates (see the electronic supplementary material, figure S1 for pre-adult host microbiome profiles). JUb19, MYb71 and BIGb0170 had high relative abundance while JUb134 was less abundant in the community (electronic supplementary material, figure S1), indicating stronger colonization abilities for the former and poorer for the latter, consistent with previous findings [24]. We did not find that bacterial colonization abilities (the fraction of relative abundance) were dependent on their placement on the phylogenetic tree (electronic supplementary material, figure S9a).

### (a) Temporal dynamics of infected and uninfected host microbiomes

**Figure 2.**
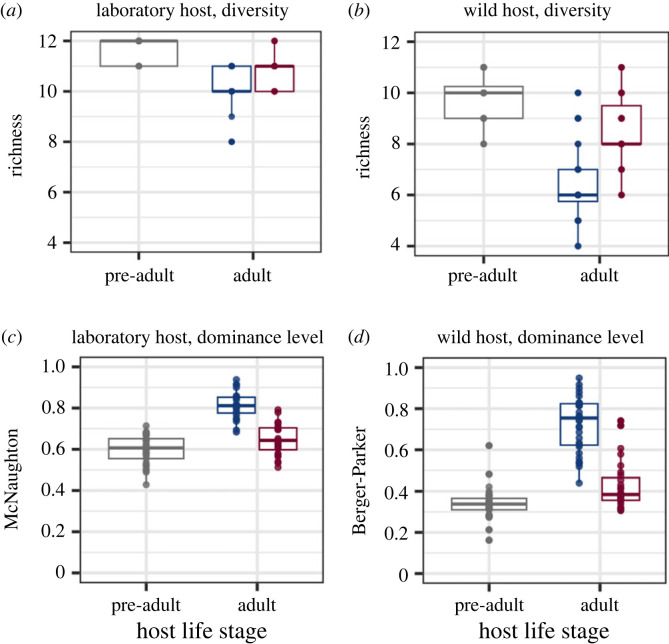
Temporal dynamics of microbiome diversity and dominance levels, across host life stages, host type, and infection treatments. (*a*) Microbiome diversity (richness) in infected and uninfected laboratory-adapted hosts over time. (*b*) Microbiome diversity (richness) in infected and uninfected wild hosts over time. (*c*) Microbiome dominance (McNaughton's index) in infected and uninfected laboratory-adapted hosts. (*d*) Microbiome dominance (Berger-Parker index) in infected and uninfected wild hosts. Significant differences are detected for all pairwise comparisons shown. Adult microbiome data are shown for infected hosts (red) and for uninfected hosts (blue).

We evaluated the temporal dynamics of host microbiomes by comparing microbiomes of pre-adult and adult hosts in the presence or absence of parasites. We found that across laboratory and wild hosts, alpha-diversity (richness and phylogenetic diversity) decreased, and dominance increased over time in both infected and uninfected hosts (figure 2). In uninfected hosts, microbiome diversity was reduced more than that in infected hosts (figure 2*a,b*; see the electronic supplementary material, figure S2 for other diversity metrics, also table S3).

We found that microbiome composition also changed over time in both uninfected and infected hosts (electronic supplementary material, table S3). For uninfected adult hosts, despite being lower in relative abundance early on, MYb10 dominated in laboratory-adapted host microbiomes, and BIGb0393 dominated in wild host microbiomes (electronic supplementary material, figure S10b). BIGb0393 had relatively higher mean metabolic interaction potential with other taxa. This pattern might be associated with an increase in the relative fitness of this species over a temporal scale (electronic supplementary material, Methods and Results). For infected hosts, increased dominance was less common over time. We observed the enrichment of BIGb0393 in wild infected adults, and BIGb0172 and BIGb0170 in laboratory-adapted infected adults (electronic supplementary material, figure S10b), but these enriched species did not widely dominate the microbiome communities (electronic supplementary material, figure S1). We found that across laboratory and wild hosts, microbiome dispersion increased over time, and much more so during infection (electronic supplementary material, table S3).

### (b) Infection increased host microbiome diversity and decreased dominance

We found that compared with uninfected adult hosts, infected hosts harboured significantly higher microbiome diversity and lower dominance across laboratory and wild isolates. This pattern was consistent across alpha diversity and dominance metrics (N2: figure 2*a,b*, table 1; wild: figure 2*c,d*, table 1; see the electronic supplementary material, figure S2 and figure S3 for other metrics). We did not observe significant associations between wild isolate latitude of origin and microbiome diversity or dominance, in either infected or uninfected controls (electronic supplementary material, table S2: *p* > 0.096 for all correlation tests). Latitude of origin also did not play a role in pre-adult host microbiome diversity or dominance (electronic supplementary material, table S3: *p* > 0.4 for all correlation tests).

**Table 1.** Comparisons of microbiome diversity, dominance and dispersion between infected and uninfected adults (uninfected hosts served as the reference in statistical tests). (*p < 0.05; **p < 0.01; ***p < 0.001.)

factor	metric	statistical test	*W*	estimate intercept	significance level	host
alpha-diversity						
infected versus uninfected	richness	Wilcoxon rank-sum test	187	n/a	*p* = 0.021*	laboratory-adapted
	Shannon	*t*-test	n/a	0.37	*p* < 0.001***	laboratory-adapted
	evenness	*t*-test	n/a	0.15	*p* < 0.001***	laboratory-adapted
	richness	Wilcoxon rank-sum test	122.5	n/a	*p* < 0.001***	wild isolates
	Shannon	Wilcoxon rank-sum test	31	n/a	*p* < 0.001***	wild isolates
	Faith's PD	Wilcoxon rank-sum test	155	n/a	*p* < 0.001***	wild isolates
	evenness	Wilcoxon rank-sum test	47	n/a	*p* < 0.001***	wild isolates
dominance level						
infected versus uninfected	Berger-Parker index	Wilcoxon rank-sum test	539.5	n/a	*p* < 0.001***	laboratory-adapted
	McNaughton's index	*t*-test	n/a	−0.16	*p* < 0.001***	laboratory-adapted
	Simpson's index	Wilcoxon rank-sum test	544	n/a	*p* < 0.001***	laboratory-adapted
	Berger-Parker index	Wilcoxon rank-sum test	817.5	n/a	*p* < 0.001***	wild isolates
	McNaughton's index	Wilcoxon rank-sum test	834.5	n/a	*p* < 0.001***	wild isolates
	Simpson's index	Wilcoxon rank-sum test	825	n/a	*p* < 0.001***	wild isolates
microbiome dispersion						
factor	metric	statistical test	*W*	significance level	host	
infected versus uninfected	Bray-Curtis	Wilcoxon rank-sum test	136	*p* < 0.001***	laboratory-adapted	
	weighted UniFrac	Wilcoxon rank-sum test	220	*p* < 0.001***	laboratory-adapted	
	Aitchison	Wilcoxon rank-sum test	244	*p =* 0.002**	laboratory-adapted	
	Bray-Curtis	Wilcoxon rank-sum test	130.5	*p* = 0.04*	wild isolates	
	weighted UniFrac	Wilcoxon rank-sum test	130	*p* = 0.042*	wild isolates	
	Aitchison	Wilcoxon rank-sum test	69	*p* < 0.001***	wild isolates	

### (c) Warming has a distinct impact on microbiome diversity despite infection

For pre-adult wild hosts, we found that developmental warming was associated with decreased microbial richness and phylogenetic diversity, compared with ambient developmental temperatures (electronic supplementary material, figure S4; richness W = 51, *p* = 0.032; Faith's PD W = 56.5, *p* = 0.009). We did not observe similar effects of developmental temperature on laboratory-adapted larval host microbiomes (electronic supplementary material, figure S5, table S11; *p* > 0.08 for all metrics).

**Figure 3.**
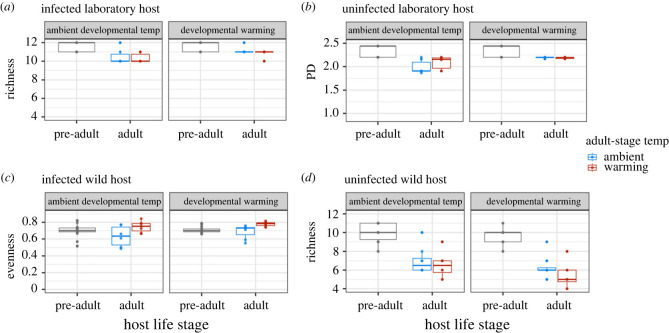
Microbiome diversity across host life stages, host type, infection treatments and warming regimes. (*a*) Microbiome richness in infected laboratory-adapted hosts over time. (*b*) Microbiome phylogenetic diversity in uninfected laboratory-adapted hosts over time. (*c*) Microbiome evenness in infected wild hosts. (*d*) Microbiome richness in uninfected wild hosts. Significant differences are detected for pairwise comparisons made in adult hosts.

Developmental temperature had consistently long-lasting effects on adult host microbiomes, regardless of parasite infection. Developmental warming was shown to increase microbiome richness and phylogenetic diversity for infected N2 hosts (figure 3*a*; richness W = 33.5, *p* = 0.014; Faith's PD W = 24.5, *p* = 0.003; see the electronic supplementary material, figure S5 for other diversity metrics) but did not change microbiome diversity for infected wild hosts (electronic supplementary material, figure S4b, table S11; *p* > 0.375 for all metrics). In uninfected N2 hosts, developmental warming increased microbiome phylogenetic diversity (figure 3*b*; Faith's PD W = 26.5, *p* = 0.008). By contrast, for uninfected wild hosts, developmental warming decreased microbiome richness (figure 3*d*; richness W = 178.5, *p* = 0.049) and increased dominance (electronic supplementary material, figure S6a; McNaughton W = 63, *p* = 0.015).

Warming during adulthood also affected microbiome diversity. Compared with ambient temperature, we found that warming increased microbiome evenness during infection in wild adults (figure 3*c*; evenness W = 29, *p* = 0.002). In the absence of parasites, warming temperatures decreased phylogenetic diversity (electronic supplementary material, figure S4a; Faith's PD W = 185.5, *p* = 0.028). By contrast, for uninfected laboratory-adapted hosts, warming during the adult stage increased microbiome Shannon diversity and evenness (electronic supplementary material, figure S5a; Shannon 25°C estimate intercept (es) = 0.195, *p* = 0.005; evenness 25°C es = 0.078, *p* = 0.005), but decreased dominance (electronic supplementary material, figure S7a; Berger-Parker Index W = 115, *p* = 0.012; McNaughton's index 25°C es = −0.07, *p* = 0.006; Simpson's index W = 120, *p* = 0.005). We did not find significant effects of warming during infection on microbiome diversity or dominance, for infected laboratory-adapted hosts (electronic supplementary material, figure S5b, figure S7b, table S11).

### (d) Developmental warming alleviated the disruptive effect of parasites

**Figure 4.**
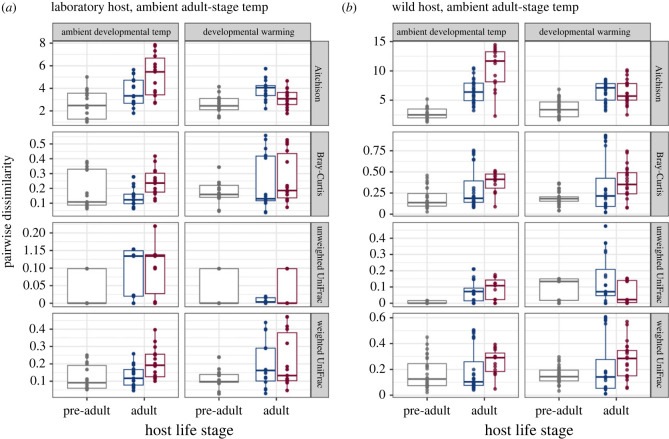
The impact of infection and developmental temperatures on host microbiome dispersion. (*a*) Microbiome dispersion for laboratory-adapted pre-adults and adults. (*b*) Microbiome dispersion for wild pre-adult and adult hosts. In both (*a*) and (*b*), adult hosts are under ambient adult-stage temperature, and grouped by different infection treatments. Plots were faceted by four beta-diversity metrics, and each data point represents a pairwise distance of two samples within the same treatment group. Adult microbiome data are shown for infected hosts (red) and for uninfected hosts (blue). Significant differences are detected in infected versus uninfected adults by Aitchison, Bray-Curtis and weighted UniFrac metrics, at ambient developmental temperatures, across laboratory and wild hosts.

Parasite infection significantly altered host microbiome composition and increased the relative abundance of rare species, such as CEent1 (electronic supplementary material, table S3, figure S10a). We investigated inter-individual microbiome variation for infected and uninfected hosts, calculated as the pairwise dissimilarity of microbiome communities. This type of variation can be used to assess microbiome dispersion in hosts under the respective treatment, with more dissimilar microbial communities indicating higher dispersion. We found that under ambient temperatures, throughout host development and adult-stage, the pairwise dissimilarity of microbial communities was significantly higher within infected adults than within uninfected adults. This pattern was consistent across laboratory-adapted and wild hosts (figure 4 and table 1).

We found that compared with ambient developmental temperature, developmental warming significantly decreased microbiome dispersion for infected wild hosts, a finding consistent across all dissimilarity metrics (figure 4*b* and table 2). For uninfected hosts, developmental warming impacted host microbiome dispersion differently for wild and laboratory-adapted hosts (figure 4). These results were inconsistent across different dissimilarity metrics. For example, we found that developmental warming increased microbiome dispersion for uninfected laboratory-adapted hosts (figure 4*a*; Aitchison W = 7052, *p* = 0.0075; weighted UniFrac W = 7144, *p* = 0.0115). Other metrics showed that developmental warming decreased microbiome dispersion for this host type (figure 4*a*; unweighted UniFrac W = 14142, *p* < 0.001). For uninfected wild hosts, developmental warming was shown to significantly decrease microbiome dispersion (figure 4*b*; Aitchison W = 9267, *p* < 0.001). Developmental warming also significantly altered laboratory-adapted host microbiome composition, whereby non-dominant species (BIGb0170) shuffled in relative abundance (table 2; electronic supplementary material, figure S1).

**Table 2.** Comparisons of microbiome beta-diversity and dispersion between hosts of different ages under different developmental temperatures. (*p < 0.05; **p < 0.01; ***p < 0.001.)

beta-diversity						
factor	metric	statistical test	pseudo-*F*	*R*^2^	significance level	host
developmental temperature for adult host (20°C versus 25°C)						
	Bray-Curtis	PERMANOVA	12.3	0.139	*p* < 0.001***	laboratory-adapted
	weighted UniFrac	PERMANOVA	19.83	0.242	*p* < 0.001***	laboratory-adapted
	Aitchison	PERMANOVA	5.24	0.033	*p* = 0.019*	laboratory-adapted
	unweighted UniFrac	PERMANOVA	21.58	0.327	*p* < 0.001***	laboratory-adapted
microbiome dispersion						
factor	metric	statistical test	*W*	significance level	host	
developmental temperature for pre-adult host (20°C versus 25°C)						
	Aitchison	Wilcoxon rank-sum test	266	*p* = 0.03*	wild isolates	
	unweighted UniFrac	Wilcoxon rank-sum test	126.5	*p* < 0.001***	wild isolates	
developmental temperature for adult host (20°C versus 25°C)						
infected						
	Bray-Curtis	Wilcoxon rank-sum test	4429	*p* = 0.006**	wild isolates	
	weighted UniFrac	Wilcoxon rank-sum test	4531	*p* = 0.002**	wild isolates	
	Aitchison	Wilcoxon rank-sum test	5264	*p* < 0.001***	wild isolates	
	unweighted UniFrac	Wilcoxon rank-sum test	4670.5	*p* < 0.001***	wild isolates	

We also assessed the effect of developmental warming on pre-adult host microbiomes. We found that developmental warming significantly altered microbial community composition (PERMANOVA unweighted UniFrac pseudo-*F* = 10.27, *R*^2^ = 0.44, *p* = 0.025) and increased microbiome dispersion for wild pre-adults (figure 4*b* and table 2). We did not observe similar effects of developmental temperature on lab-adapted larval host microbiomes (figure 4*a*; electronic supplementary material, table S11).

### (e) Infection and warming during adulthood increased microbiome dispersion in a non-additive fashion

We found that warming during adulthood significantly increased host microbiome dispersion in uninfected laboratory-adapted and wild adults, compared with ambient adult-stage temperatures (electronic supplementary material, figure S8, table S11; laboratory-adapted host: Aitchison *W* = 6264, *p* < 0.001; wild isolates: unweighted UniFrac *W* = 3405, *p* < 0.001). This destabilizing effect was similar to that observed for infection. We saw that warming during infection did not increase microbiome dispersion additively in laboratory-adapted hosts (electronic supplementary material, figure S8a, table S11; *p* > 0.067 for all metrics). However, these factors together reduced dispersion in microbiomes of infected wild hosts (figure 4; electronic supplementary material, figure S8b; Bray-Curtis *W* = 5941.5, *p* < 0.001; weighted UniFrac *W* = 5484, *p* < 0.001).

### (f) Host microbiome dispersion is associated with less variable species interactions

**Figure 5.**
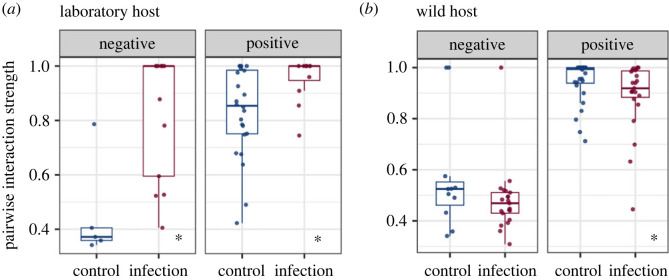
The impact of parasite infection and host type on the strength and variation of species interactions within microbiomes. (*a*) Infection impacted the strength of positive and negative interactions, and the variation of positive interaction strength, in the laboratory-adapted hosts. (*b*) Infection impacted the strength of positive interactions in wild hosts. The *y*-axis for both (*a*) and (*b*) represents the absolute value of species co-occurrence coefficient. Adult microbiome data are shown for infected hosts (red) and for uninfected hosts (blue). Asterisks indicate significant comparisons between groups on interaction strength.

We investigated how warming and infection could influence species interaction strength in host microbiome communities. For laboratory-adapted adult hosts, we found that parasite infection significantly strengthened both positive and negative interactions but decreased the variation in interaction strength (figure 5*a*; Wilcoxon rank-sum test, positive links: *W* = 68, *p* = 0.01; negative links: *W* = 5, *p* = 0.006; Levene's test: positive links *F* = 5.87, *p* = 0.021; all links *F* = 17.49, *p* < 0.001). Similar effects of lowered variation in interaction strength were found for wild adult host microbiomes over time, in the absence of infection (Levene's test *F* = 4.14, *p* = 0.046). For wild adult hosts, we found that infection slightly decreased the strength of positive interactions (figure 5*b*; Wilcoxon rank-sum test, *W* = 452, *p* = 0.013). We did not observe significant effects of warming on species interactions strength in either laboratory or wild host microbiomes (electronic supplementary material, table S11; *p* > 0.05 for all comparisons).

## 4. Discussion

Climate change and infectious diseases have led to population declines of animals and plants thereby threatening ecosystem biodiversity [1–4]. Host-associated microbial communities have the potential to rapidly respond to biotic and abiotic disturbance, thus providing meaningful early indicators of ecosystem and host health [14,16,55]. Studying the general response of resident microbiomes to temperature changes and infection together could shed light on the persistence of host species in a changing world.

We found that parasite infection significantly altered host microbiome dynamics, with the effects similar to the removal of dominant competitors in the community [56]. These effects could be driven by parasite-induced alteration in inter-species competition and cooperation [57]. We observed a gradual loss of microbiome diversity and increased dominance level over time; changes in these metrics were more prominent in uninfected hosts compared with infected hosts. Across temporal scales, we found that some bacterial species (potentially stronger competitors) dominated uninfected host microbiomes. Parasite infection reduced dominance of these taxa, leading to a more diverse and even community over time. Parasite-induced disturbance has been shown to drive microbial communities to alternative stable states [58], with shifts in the abundance of specific taxa [59,60].

**Figure 6.**
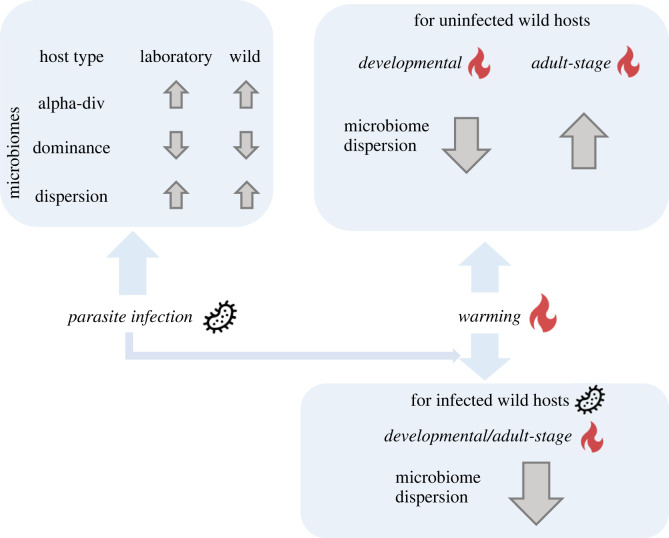
Schematic of main findings in microbiome changes by infection and warming regimes. Top left panel: parasite infection impacts microbiome alpha-diversity, dominance level and dispersion consistently for laboratory-adapted N2 (laboratory) and wild isolates (wild). Top right panel: warming at different host life stages has distinct impact on microbiome dispersion for uninfected wild hosts. Bottom right panel: warming and infection act in an opposing way on microbiome dispersion for wild hosts. Up or down arrows indicate significant increases or decreases of the relevant microbiome measures.

We found that infection was associated with higher microbiome diversity and lower dominance level (figure 6). More widely, the direction in which the parasite alters gut microbiome diversity varies depending on the species and context [59–61]. Infection is associated with increased microbiome alpha diversity in some systems [59,62,63] and decreased diversity in others [64–67]. Whilst the impact of parasites on microbiome dynamics varies across systems [62–67], increased alpha diversity under infection can be caused by an altered gut immune environment with prominent inflammation [59,62,68]. We show that warming impacts host microbiome diversity less prominently than infection, with the effects differing by timing of warming as well as the degree of laboratory adaptation by the hosts.

Here, infection drove larger changes in microbiome composition than warming. Previous work has shown both temperature and infection change amphibian skin microbiome structure, with temperature effects probably mediated via reductions in parasite load [13]. We revealed that parasite infection and warming, independently and simultaneously destabilized host microbiomes, as shown by an increase in dispersion ([28,69,70]; figure 6). Destabilization can suggest that the stressed host is less able to regulate its microbiome community [28–30]. This increase in dispersion caused by both abiotic and biotic stressors supports the Anna Karenina principle, adapted and used to predict consequences for animal microbiomes under dysbiosis [28]. The principal proposes that dispersed microbiomes are more likely to occur in stressed individuals than healthy individuals, as found in disease-associated human and animal gut microbiomes [29,30,71,72]. Such infection- or warming-induced Anna Karenina effects have been documented in microbiomes hosted by multiple animal species [28,73,74] and captured in long-term field experiments [75].

The level of destabilization caused by infection was the same or lower as when warming was added on top (figure 6). Stressors can be temporally and spatially variable in nature, but also occur simultaneously, with the synergy between multiple stressors thought to accelerate biodiversity loss [76]. Previous studies showed that multiple stressors (i.e. nutrient pollution [75], simulated predation [77], overfishing [75] and warming [77]) of coral microbiomes acted in ‘opposing’, rather than a synergistic or additive fashion [75,77]. We found similar results in nematode microbiomes herein. Our results from wild nematode microbiomes showed that multiple stressors generate less dispersion compared to that caused by single stressors. However, the timing of those stressors, as well as the degree of laboratory adaptation by the host, are important factors for shaping the interactive outcome of those multiple stressors.

The timing of warming during the host's life-cycle, specifically during development, had a large impact on wild host microbiomes (figure 6). Compared with ambient developmental temperature, developmental warming alone increased microbiome dispersion in wild larval hosts. The timing of warming alleviated the microbiome instability caused by infection. Hosts at different life stages could vary in their sensitivity to warming [78]. Heat stress during nematode larval stages can activate heat shock transcription factors and protein production, which is part of the multi-pathogen defence pathways of *C. elegans* [79,80]. A similar protective effect of early-stage heat exposure has been found for broiler chickens [81] and plant hosts (e.g. *Arabidopsis thaliana*) [82,83]. Early heat exposure can also protect hosts against heat stress later on in life [84]. The induced resistance by early environmental stress (e.g. chemical agents [85], physical wounding [86]) is widely applied in plant hosts to increase their basal resistance to future attacks [87]. Periods of heat stress during climate change should be investigated further as a driver of infection patterns and epidemiology in animals as they age.

We found that parasite infection induced stronger species interactions (both competitive and facilitative) in laboratory-adapted host microbiomes. This finding might be a signature of poor host control [70,88]. Bacterial communities are shaped by inter-species interactions, such as competition for shared resources and facilitation by metabolite exchanges [89]. Predictions of species competition can benefit from genome-scale metabolic network construction and inference of species metabolic resource overlap [90]. We revealed that species competition strength could also be negatively associated with their phylogenetic relatedness, supported by previous findings [91]. Microbial species interactions can have large impacts on host fitness [92]. So, these results highlight the importance of applying microbial community ecology to understand host-microbe interactions under changing environments. Our results from laboratory-adapted hosts revealed that increased microbiome dispersion induced by infection was associated with stronger interaction strength in the community, reinforcing the hypothesis that strong interactions could lead to unstable community dynamics [93–96]. We also showed that higher microbiome dispersion induced by infection, or over time without infection, was associated with lower variation in species interaction strength, in laboratory-adapted and wild nematode hosts, respectively. Higher variation of interaction strength could confer resilience and stability for communities [97,98]. This hypothesis remains untested on microbial communities.

Amidst climate change, hosts and their microbiomes are commonly exposed to stressful temperatures. Microbiome changes, particularly patterns of dispersion and instability, have been linked with animal host health [14,28,30,59]. Assessing microbiome structure and dynamics in response to multiple stressors (separately and together) across time will probably be important in predicting host population persistence in the wild as climate change progresses. This multi-factor approach and focus on dynamics will also help to refine microbiome-based interventions against infection to conserve endangered species [99,100].

## Data Availability

16S rRNA sequencing data and associated metadata are available from the National Center for Biotechnology Information Sequence Read Archive: www.ncbi.nlm.nih.gov/sra under Bioproject ID PRJNA1002096. Electronic supplementary material is available online at figshare [101].
